# *Schistosoma japonicum* transmission risk maps at present and under climate change in mainland China

**DOI:** 10.1371/journal.pntd.0006021

**Published:** 2017-10-17

**Authors:** Gengping Zhu, Jingyu Fan, A. Townsend Peterson

**Affiliations:** 1 Tianjin Key Laboratory of Animal and Plant Resistance, College of Life Sciences, Tianjin Normal University, Tianjin, China; 2 Biodiversity Institute, University of Kansas, Lawrence, Kansas, United States of America; George Washington University, UNITED STATES

## Abstract

**Background:**

The South-to-North Water Diversion (SNWD) project is designed to channel fresh water from the Yangtze River north to more industrialized parts of China. An important question is whether future climate change and dispersal via the SNWD may synergistically favor a northward expansion of species involved in hosting and transmitting schistosomiasis in China, specifically the intermediate host, *Oncomelania hupensis*.

**Methodology/ Principal findings:**

In this study, climate spaces occupied by the four subspecies of *O*. *hupensis* (*O*. *h*. *hupensis*, *O*. *h*. *robertsoni*, *O*. *h*. *guangxiensis* and *O*. *h*. *tangi*) were estimated, and niche conservatism tested among each pair of subspecies. Fine-tuned Maxent (*f*Maxent) and ensemble models were used to anticipate potential distributions of *O*. *hupensis* under future climate change scenarios. We were largely unable to reject the null hypothesis that climatic niches are conserved among the four subspecies, so factors other than climate appear to account for the divergence of *O*. *hupensis* populations across mainland China. Both model approaches indicated increased suitability and range expansion in *O*. *h*. *hupensis* in the future; an eastward and northward shift in *O*. *h*. *robertsioni* and *O*. *h*. *guangxiensis*, respectively; and relative distributional stability in *O*. *h*. *gangi*.

**Conclusions/Significance:**

The southern parts of the Central Route of SNWD will coincide with suitable areas for *O*. *h*. *hupensis* in 2050–2060; its suitable areas will also expand northward along the southern parts of the Eastern Route by 2080–2090. Our results call for rigorous monitoring and surveillance of schistosomiasis along the southern Central Route and Eastern Route of the SNWD in a future, warmer China.

## Author summary

The South-to-North Water Diversion (SNWD) project is designed to channel fresh water from the Yangtze River north to more industrialized parts of China. An important question is whether future climate change and dispersal via the SNWD may synergistically favor northward expansion of schistosomiasis in China. Our models indicated increased suitability and range expansion in *Oncomelania h*. *hupensis* in the future; an eastward and northward shift in *O*. *h*. *robertsioni* and *O*. *h*. *guangxiensis*, respectively; and relative stability in *O*. *h*. *gangi*. The southern Central Route of SNWD will coincide with suitable areas for *O*. *h*. *hupensis* in 2050–2060; its suitable areas will also expand northward along the southern Eastern Route in 2080–2090. Our results call for rigorous monitoring and surveillance of schistosomiasis along the southern Central Route and Eastern Route of the SNWD in a future, warmer China.

## Introduction

Schistosomiasis is a neglected tropical disease that is known to have affected people in China for more than 2100 years, with presently ~800,000 infected and ~65 million people at risk of infection [1]. The challenge of combatting this disease lies in the wide distribution of its snail hosts and the broad range of domestic and wild mammals that act as reservoirs for human infections [2]. Chinese schistosomiasis is caused by the digenetic blood trematode *Schistosoma japonicum*, a parasitic flatworm that completes its life cycle through one intermediate (i.e. the snail *Oncomelania hupensis*) and diverse definitive (i.e. mammals) hosts. Over the past five decades, China has made remarkable progress in reducing *S*. *japonicum* infections in humans through a combination of chemotherapy and snail control, but schistosomiasis has re-emerged in recent years owing to changes in ecological and socio-economic factors, together with termination of the World Bank Loan Project on schistosomiasis control in 2001 [3]. Given that schistosomiasis is unlikely to be eliminated, considering whether and how future climates are likely to impact its transmission becomes increasingly important.

Based on the environmental variables that associated with species’ occurrence records, ecological niche modeling (ENM) seeks to characterize environmental conditions suitable (i.e. realized niche) for a particular species and then identify where suitable environmental habitats are distributed in the space [4], it is a powerful tool in studies of effects of global climate change on the geography of disease transmission [5]. Assumptions under which ENMs work best include equilibrium between species’ distributions and their ecological requirements, and conservatism of ecological niche [4]. Among them, niche conservatism providing support for using ENMs has been widely noticed, the degree to which plants and animals retain their ancestral ecological traits and environmental distributions ('niche conservatism') is hotly debated, in part because of its relevance to the fate of modern species facing climate change [6].

ENM tools, however, are also subject to issues including the need to balance goodness-of-fit against model complexity [7], and the importance of considering uncertainty in model predictions [8]. These issues are particularly critical in studies involving transfer of models across space or time (e.g. climate change effects). Recent efforts have developed methods to reduce model complexity and characterize uncertainty, and thereby improve model transferability in forecasting climate change effects [9–12]. These steps include species-specific tuning of settings (rather than default setting) to improve model performance [9,10], evaluation using spatially independent training and testing data sets [12], and integrating multiple predictions via ensemble approaches [11,12].

*Oncomelania hupensis* is the sole intermediate snail host of *S*. *japonicum* in China, which thus depends entirely on this snail species for transmission [13]. However, the taxonomy of *O*. *hupensis* in mainland China has been debated in view of marked morphological variation. Liu et al. recognized 5 subspecies [14], whereas Davis et al. treated only 3 subspecies based on shell form, allozyme data, and biogeography [15]. However, Zhou et al. separated *O*. *h*. *guangxiensis* from *O*. *h*. *hupensis* based on molecular characters, and recognized 4 subspecies in mainland China [16], which was later verified by Li et al. based on internal transcribed spacer (ITS) and 16S fragments [17,18]. Here, we consider the four subspecies [14,16,18,19]; at present, *O*. *h*. *hupensis* and *O*. *h*. *robertsoni* dominate transmission of *S*. *japonicum*, as control measures have reduced *O*. *h*. *guangxiensis* and *O*. *h*. *tangi* considerably [13]. These four subspecies differ in shell size and structure, breeding environment, growth rates, population genetics, and potential for infection by *S*. *japonicum* [17].

Previous attempts to predict spatial dimensions of transmission risk of schistosomiasis have characterized transmission environments of *S*. *japonicum* [20–22] or ecological requirements of *O*. *hupensi* [23,24]; these studies were generally conducted at local geographic scales and with limited temporal coverage. Several environmental correlates of *S*. *japonicum* transmission have been identified, including distance to snail habitat and wetlands, seasonal land surface temperature, and seasonal variation of vegetation indices [21,22]. Climate conditions explain much variation in transmission of schistosomiasis, especially at regional and continental scales [25,26]. Understanding ecological dimensions and potential distribution of O. *hupensis* is thus crucial [20], and yet has not seen detailed analysis.

**Fig 1:**
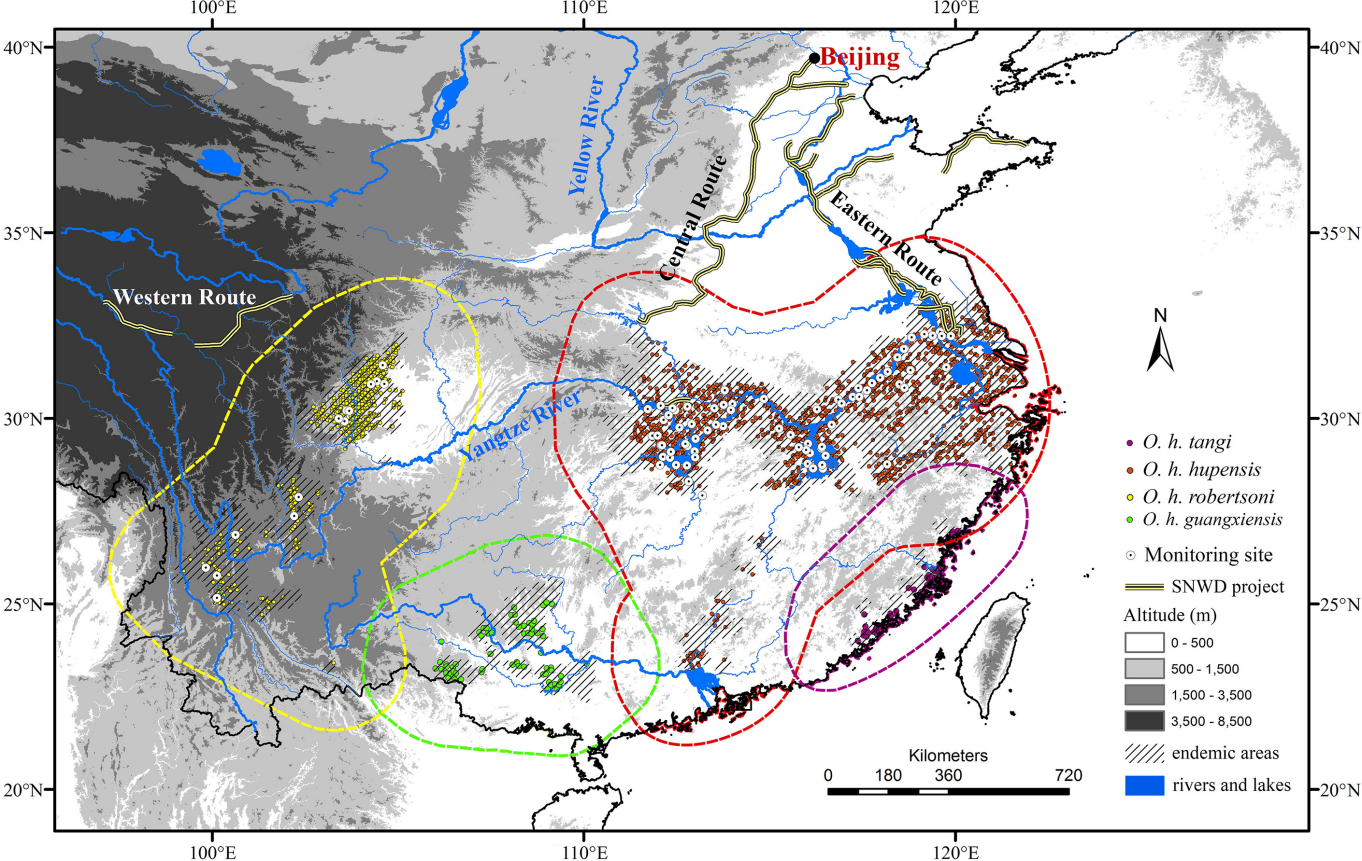
Geographic distribution of the four subspecies of *Oncomelania hupensis* in mainland China. Dashed color lines denote the accessible areas used for climate space comparison and niche modeling. Three routes of South-to-North-Water-Diversion project, surveillance sites, occurrence data, and administrative endemic areas are overlaid on elevation as a background.

The South-to-North Water Diversion (SNWD) project is a multi-decade mega-project in China. It is the biggest inter-basin transfer scheme in the world, aiming to channel 25 × 10^9^ m^3^ fresh water annually from the Yangtze River in southern China to the more arid and industrialized north via two routes (i.e. the Central Route and Eastern Route, Fig 1). In the context of climate change, in which the geographic potential of *O*. *hupensis* may change, the relationship of such changes to planned SNWD corridors remains unknown. Surveillance sites were established during 2002–2010 across mainland China (Fig 1); however, most sites were located along the Yangtze River at low elevations, focused on transmission by *O*. *h*. *hupensis* and *O*. *h*. *robertsoni*. The questions of whether future climate change and the SNWD project may synergistically favor expansion of some population of *O*. *hupensis*, and whether the existing surveillance sites are sufficient, necessitate the present study.

In this study, we used a unique dataset of *O*. *hupensis* presences from more than 5 thousand villages to explore ecological dimensions and potential distributions of *O*. *hupensis* in mainland China. The aims of this study were to (1) compare climate spaces occupied by the four subspecies of *O*. *hupensis*, to (2) test whether climate niches were conserved during the four subspecies’ divergence (i.e. climate niche conservatism evaluation), to (3) predict their potential distributions using state-of-the-art modelling techniques, to (4) investigate the potential impacts of future climate change and the SNWD project on *O*. *hupensis*. The overall purpose was to predict the *S*. *japonicum* transmission risk at present and under climate change in mainland China.

## Methodology

### Data collection

Occurrence data for subspecies of *O*. *hupensis* were assembled from Qian [27]. This national surveillance effort of schistosomiasis was carried out at the village level between the 1950s and 1980s across 12 Chinese provinces. In all, 5029 towns and villages reported presence of *O*. *hupensis* [27]. Rather than using centroids of infested counties, which reduces precision, we georeferenced individual villages using Google Maps. These points varied in terms of clumping, so we subsampled them to reduce sampling bias and spatial autocorrelation [28], as follows. First, we arranged infested provinces according to sample density (i.e. number of occurrence points divided by area of the province). The median served as the standard sampling effort, and all provinces presenting densities above that value were subsampled randomly to a lower density. In the end, we had 1996 occurrence points: 1402 *O*. *h*. *hupensis*, 470 *O*. *h*. *robertsoni*, 64 *O*. *h*. *guangxiensis*, and 60 *O*. *h*. *tangi* (S1 File).

Several approaches have been used to select environmental datasets for ecological niche modeling; the best environmental datasets would be ecological relevant to species in question [29]. At regional and continental scales, climatic factors have excellent predictive power in determining risk associated with disease transmission (e.g. schistosomiasis [25,26], West Nile virus [30]). Hence, we used subsets of the 19 bioclimatic variables developed by Hijmans et al. [31], chosen as follow. First, variables that combined temperature and precipitation (i.e. mean temperature of wettest quarter, mean temperature of driest quarter, precipitation of warmest quarter, precipitation of coldest quarter) were excluded because they display artificial discontinuities between adjacent grid cells in some areas [32]. The importance of each of the remaining 15 variables was assessed by a jackknife analysis of variable importance in Maxent ([33], see below for Maxent detail), and unimportant variables were discarded. Highly correlated variables were then removed in SDMtoolbox, a python-based GIS toolkit for spatial analysis [34]. Eight variables (S1 Table) that showed ecological relevance (regularized training gain >0.14) and low correlation with other variables (Pearson correlation <0.9) were chosen in the end. All variables were analyzed at a spatial resolution of 2.5 minute.

### Climate space comparison

Climatic spaces occupied by the four subspecies were first compared along each environmental dimensions using violin plots, which combine the functions of boxplot and kernel density, providing a better indication of the shape of the data distribution. We used NicheA, a toolkit to create and visualized ecological niches in environmental spaces [35], to visualize climate niches occupied by each subspecies in reduced multiple environmental spaces: we displayed the first three principal components derived from the 8 bioclimatic layers, and plotted minimum volume ellipsoids (MVEs) around occupied conditions. We quantified niche overlap between pairs of subspecies using Schoener’s *D* [36]; this metric ranges from 0 (no overlap) to 1 (complete overlap), and was used to test niche identity and niche similarity between subspecies. Niche identity and similarity tests were performed to determine whether climate spaces occupied by the two subspecies were identical or exhibited significant difference, and whether these differences were caused by the environmental feature spaces [37]. Niche identity was tested by randomly allocating occurrence records within each pair 500 times, according to observed numbers of records, and comparing observed and simulated Schoener’s *D* estimates. In contrast, niche similarity was tested by shifting the centroid of the observed occurrence densities to a random location within the available environmental space 500 times, and comparing observed with the null distribution of simulated estimates of Schoener’s *D* [37]; climate variables measured at locations across the available backgrounds of subspecies were combined and projected onto the first two principal components using PCA_env package [37]. Smoothed densities of occurrences and available environments in each grid cell were calculated and compared among the four subspecies [37].

Background environments for climate niche comparisons and niche model calibration should include only areas that have been accessible to the populations under study [38]. We delimited this area by buffering a convex hull around known occurrences by 200 km (Fig 1) in SDMtoolbox [34]. This approach reflects a compromise between including all environments that have been accessible to the species, and still covering a broad-enough extent to minimize extrapolation and detect climatic differences between presence and background records [39].

### Ecological niche modeling

To forecast climate change effects, we used *f*Maxent (fine-tuned Maxent, see below) and ensemble approaches [11,12] to calibrate models under present conditions, which were then transferred onto climate conditions for 2050 and 2080. Maxent is the most commonly used method in ENM, and it can fit arbitrarily complex models to explain relationships between environmental variables and occurrence data (version 3.3.3k; [40]). However, because an excessively complex model will be extremely specific to input data and perform poorly when extrapolating, Warren and Seifert proposed using a sample-size-adjusted Akaike information criterion (AICc) as criteria with which to address overfitting; this approach does not control model fit directly, but rather uses AICc to choose appropriate settings [7]. We used the “ENMeval” package [9] to fine-tune Maxent models by seeking the minimum value of AICc among candidate models. ENMeval provides an automated way to execute Maxent models across a user-specified range of regularization multiplier (RM) values and features combinations (FC). We set the RM range to 0.5–6.0 with increments of 0.5, and used 6 FCs, to cover a broad range of model settings. The block method was used to partition occurrence data into four bins, 3 of which were used for training and the remaining one for testing (bin combination, BC), which is desirable for studies involving model transferring [9]. In all, 2160 models (12 RMs × 6 FCs× 6 BCs × 5 occurrence groups) were generated for the four subspecies and for *O*. *hupensis* as a whole.

Ensemble models are used commonly in forecasting climate change effects, seeking to generate a consensus estimate that reduces individual model uncertainty by reflecting the central tendency of multiple models [11,12,41]. Here, outputs from six modelling algorithms, including generalized additive models (GAM), generalized boosted models (GBM), generalized linear models (GLM), random forests (RF), genetic algorithms (GARP), and the *f*Maxent model described above were included in ensembles. Individual GAM, GBM, GLM, and RF models were developed using BIOMOD2 [42], as implemented in R [43]; GARP models were developed in desktopGARP [44]. Details of implementation of each algorithm are provided in the supporting information (S2 Table). Model ensembles typically use a weighted averaging approach, in which models are weighted according to their interpolative performance (e.g. [30]). However, a recent assessment pointed to the challenge of balancing model interpolative accuracy against transferability [29,45]. Therefore, rather than using weighted averages, we used the PCA (median) method to identify the “central tendency” of individual model predictions [8,11]. The PCA measures, for each model, its ability to follow the general trend of predictions of the six models. This method calculates the median of the four individual models that had higher factor values among the six models [8,11,12].

The occurrence data used to fit the niche models were split randomly into two datasets, for calibration (70% of points) and interpolation evaluation (30% of points). Performance of individual and consensus models was evaluated via a partial ROC (receiver operating characteristic) approach [46]. Comparing to traditional AUC (area under the ROC curve), which was criticized because present data are more reliable than absence data in model evaluation [46], the partial ROC approach takes the quality of occurrence points into account and weights more on omission error [46]). Here, AUC calculations were limited to ROC spaces over which models actually made predictions, and only omission errors <5% were considered (i.e. *E* = 5%; [46]).

Final model runs incorporating all point data were used for visualizations and risk assessments. A modified least training presence threshold based on *E* = 5% was applied to *f*Maxent model predictions for *O*. *hupensis* and *O*. *h*. *hupensis* to generate binary predictions. We did not generate threshold predictions in ensemble future projection because such predictions are not applicable and hard to interpret (i.e. individual models for generating consensus models were different in present and future predictions).

### Future climate scenarios

Future climate variables were downloaded from WorldClim [31], the Consultative Group on International Agricultural Research (CGIAR), and the research program on Climate Change, Agriculture and Food Security (CCAFS). To reduce uncertainty regarding future climate conditions (S1 Fig), rather than using the 13 original global climate models (GCMs, S3 Table) from the IPCC 5th Assessment, the PCA (median) protocol was also used to generate consensus “climate models” among the 13 GCMs for each climate dimensions for 2050–2060 and 2080–2090 (S4 Table).

The *f*Maxent and ensemble models based on present predictions were applied to these future conditions. Future climate models applied to the intermediate scenario of representative concentration pathways of 4.5 (i.e. “RCP45”; [47]) in which future anthropogenic greenhouse gas emissions were estimated to peak around 2040. This scenario was chosen because it represents the middle range of available four scenarios, and as such is considered more realistic than models based on extremely high or extremely conservative scenarios [47]. Climatic similarities between present and future in 2050 and 2080 were assessed using mobility-oriented parity (MOP) metrics, a correction and simplification of multivariate environmental similarity surfaces [39].

## Results

### Spatial comparison

Different degrees of overlap were observed in the eight climate dimensions among the four subspecies (S2 Fig). *Oncomelania hupensis hupensis* and *O*. *h*. *robertsoni* occupied similar temperature and precipitation dimensions in terms of annual mean temperature (bio1), mean diurnal temperature range (bio2), and annual precipitation (bio12), but not isothermality (bio3), temperature seasonality (bio4), mean temperature of warmest quarter (bio10), or precipitation of driest month (bio14); *O*. *h*. *guangxiensis* and *O*. *h*. *tangi* occupied similar temperature and precipitation regimes in terms of annual mean temperature (bio1), temperature seasonality (bio4), mean temperature of warmest quarter (bio10), annual precipitation (bio12), and precipitation of driest month (bio14), but not mean diurnal temperature range (bio2) or isothermality (bio3) (S2 Fig). The four subspecies showed diverse responses to precipitation seasonality (bio15).

**Fig 2:**
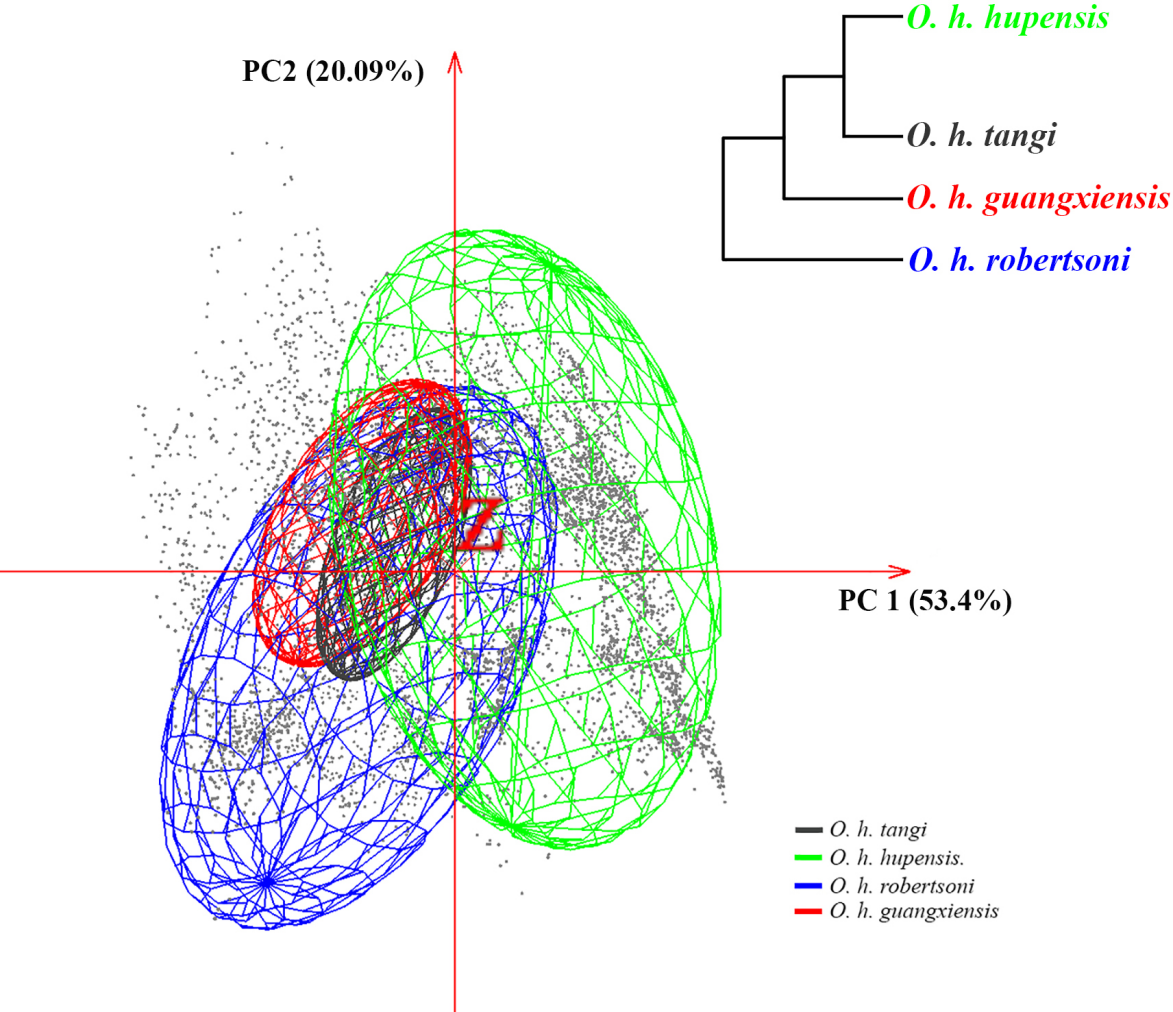
Minimum volume ellipsoids occupied by the four subspecies of *Oncomelania hupensis*. The inset shows the phylogenetic relationships of the four subspecies based on 16S sequences [18]. Gray dots denote the background conditions available to *O*. *hupensis* across mainland China.

Minimum volume ellipsoids occupied by the subspecies overlapped broadly (Fig 2). The size of the MVEs corresponded roughly to the geographic range extent of each subspecies (Figs 1 and 2), with *O*. *h*. *hupensis* and *O*. *h*. *robertsoni* occupying larger volumes than *O*. *h*. *tangi* and *O*. *h*. *guangxiensis*. Niche overlaps between pairs of subspecies also corresponded roughly to their genetic distances estimated by 16S sequence (Fig 2 and S3 Fig): i.e. the close relationship between *O*. *h*. *hupensis* and *O*. *h*. *tangi* coincided with the highest climatic niche overlap (*D* = 0.215) among all pairs (S3 Fig). Similar patterns were observed between *O*. *h*. *tangi* and *O*. *h*. *guangxiensis* (*D* = 0.147), but to a lesser extent (Fig 2 and S3 Fig). The null hypothesis of niche identity was rejected in all pairwise comparisons (S3 Fig). However, in analyses of niche similarity, the null hypothesis could not be rejected, except for *O*. *h*. *robertsoni* versus *O*. *h*. *tangi* (S3 Fig). Results of niche identity and similarity thus suggest that, although the four subspecies occupy unique climate spaces, the nonequivalence of niche spaces derives from a background effect, and not from biological differences.

### Model performance

**Fig 3:**
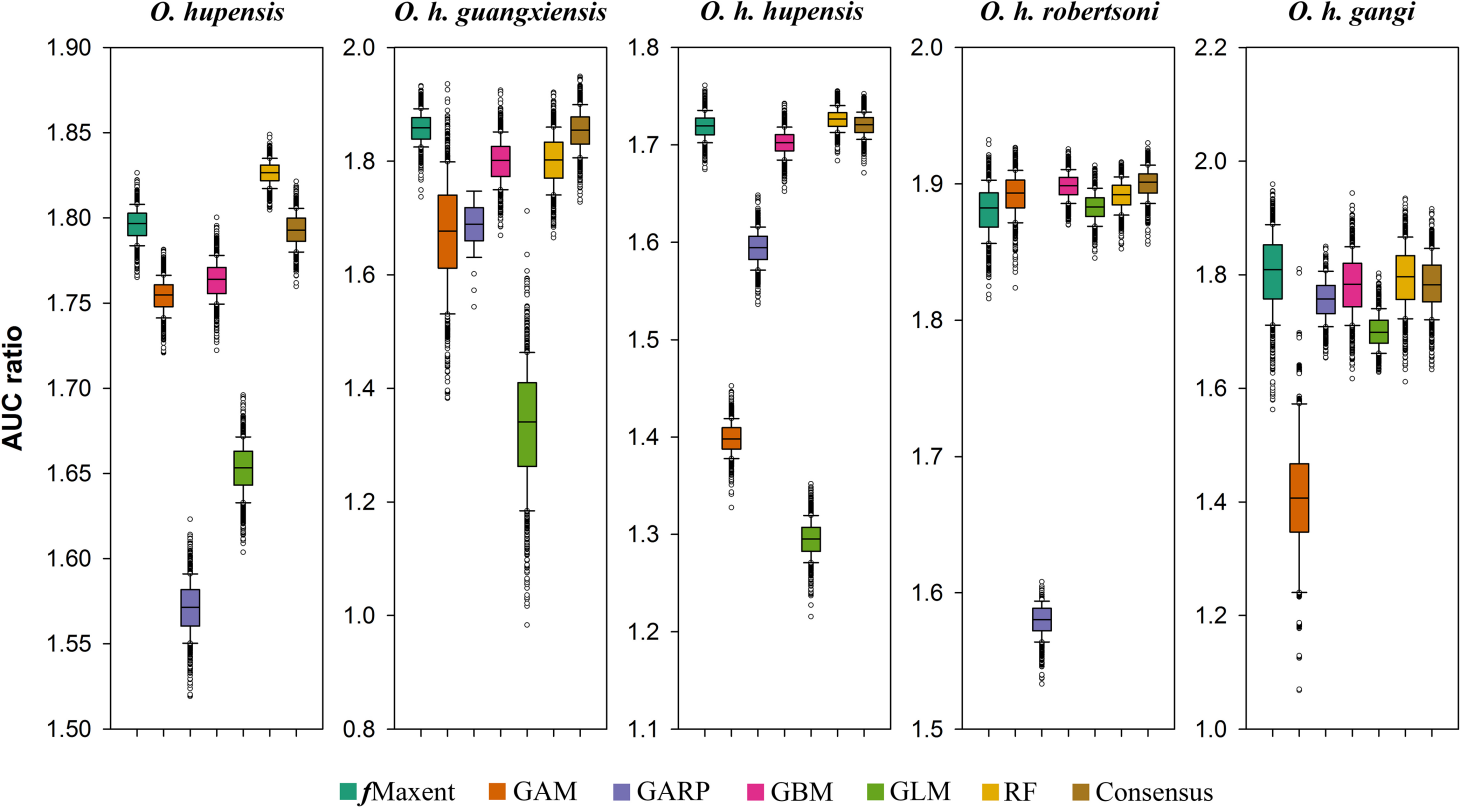
Distributions of model evaluation results (AUC ratios) of individual and consensus models in interpolation validations. Models were generated for the four subspecies separately and for *Oncomelania hupensis* as a whole.

Individual model performances varied across model algorithms and subspecies in interpolation validations (Fig 3). The machine learning methods (i.e. *f*Maxent, GBM, RF) generally showed better discriminant ability than regression models (i.e. GAM, GLM); GARP showed unstable performance (Fig 3). Similar to the machine learning models, consensus models showed good discriminant ability for the individual subspecies and for *O*. *hupensis* as a whole (Fig 3).

Using all of the occurrence data, parameters of AICc-selected models (i.e. *f*Maxent) differed from default settings (S4 Fig). Based on block partitions of occurrence data, mean AUCtest values of *f*Maxent models were 0.79, 0.79, 0.80, 0.86, and 0.94 for *O*. *hupensis* (as a whole), *O*. *h*. *guangxiensis*, *O*. *h*. *hupensis*, *O*. *h*. *robertsoni* and *O*. *h*. *tangi*, respectively, with *f*Maxent models of *O*. *h*. *hupensis* (AUCdiff = 0.09) and *O*. *h*. *tangi* (AUCdiff = 0.02) showing less overfitting than the other three (S4 Fig). In species-wide consensus models, the first principal component explained 46.7–72.2% of individual model variation (Table 1). Consensus models were discriminated by the first axis of the PCA, and each individual model was selected in consensus model processing (Table 1).

**Table 1:** Variance projection of individual models on the first component obtained from a principal component analysis (PCA) of the six individual predictions. Percentage of variance that PC1 explained, together with the factor loadings of individual models, are shown. Bold values are the four models for which the PC1s were the highest for each combination of species x time, which were selected for the “PCA(median)” consensus. Individual models were generated for the four subspecies separately and for *Oncomelania hupensis* as a whole.

	PC1 variance	Models					
Present		*f*Maxent	GAM	GARP	GBM	GLM	RF
***O*. *hupensis***	58.9%	**0.313**	0.075	**0.816**	**0.328**	0.089	**0.339**
***Q*. *h*. *guangxiensis***	53.8%	0.038	**0.904**	0.051	**0.086**	**0.399**	**0.115**
***Q*. *h*. *hupensis***	68.4%	-0.005	**0.020**	**-0.082**	0.008	**0.996**	**-0.023**
***Q*. *h*. *robertsoni***	65.6%	-0.003	0.043	**0.135**	**0.138**	**0.978**	**0.073**
***Q*. *h*. *tangi***	59.8%	-0.019	-0.002	**0.268**	**-0.216**	**0.930**	**-0.131**
**2050s**							
***O*. *hupensis***	69.1%	**0.309**	**0.461**	**0.489**	0.241	**0.568**	0.269
***Q*. *h*. *guangxiensis***	49.7%	0.091	**0.830**	0.039	**0.102**	**0.525**	**0.126**
***Q*. *h*. *hupensis***	68.5%	0.290	**0.406**	0.147	**0.336**	**0.729**	**0.293**
***Q*. *h*. *robertsoni***	72.0%	0.032	0.103	**0.207**	**0.186**	**0.948**	**0.110**
***Q*. *h*. *tangi***	65.8%	-0.006	0.009	**0.278**	**-0.205**	**0.928**	**-0.141**
**2080s**							
***O*. *hupensis***	72.2%	**0.301**	**0.503**	**0.429**	0.216	**0.601**	0.253
***Q*. *h*. *guangxiensis***	46.7%	**0.150**	**0.719**	0.048	0.141	**0.645**	**0.151**
***Q*. *h*. *hupensis***	70.5%	**0.298**	**0.465**	0.112	**0.317**	**0.716**	0.263
***Q*. *h*. *robertsoni***	69.3%	0.029	**0.103**	**0.270**	**0.149**	**0.941**	0.089
***Q*. *h*. *tangi***	70.1%	-0.003	0.011	**0.293**	**-0.193**	**0.926**	**-0.138**

**Fig 4:**
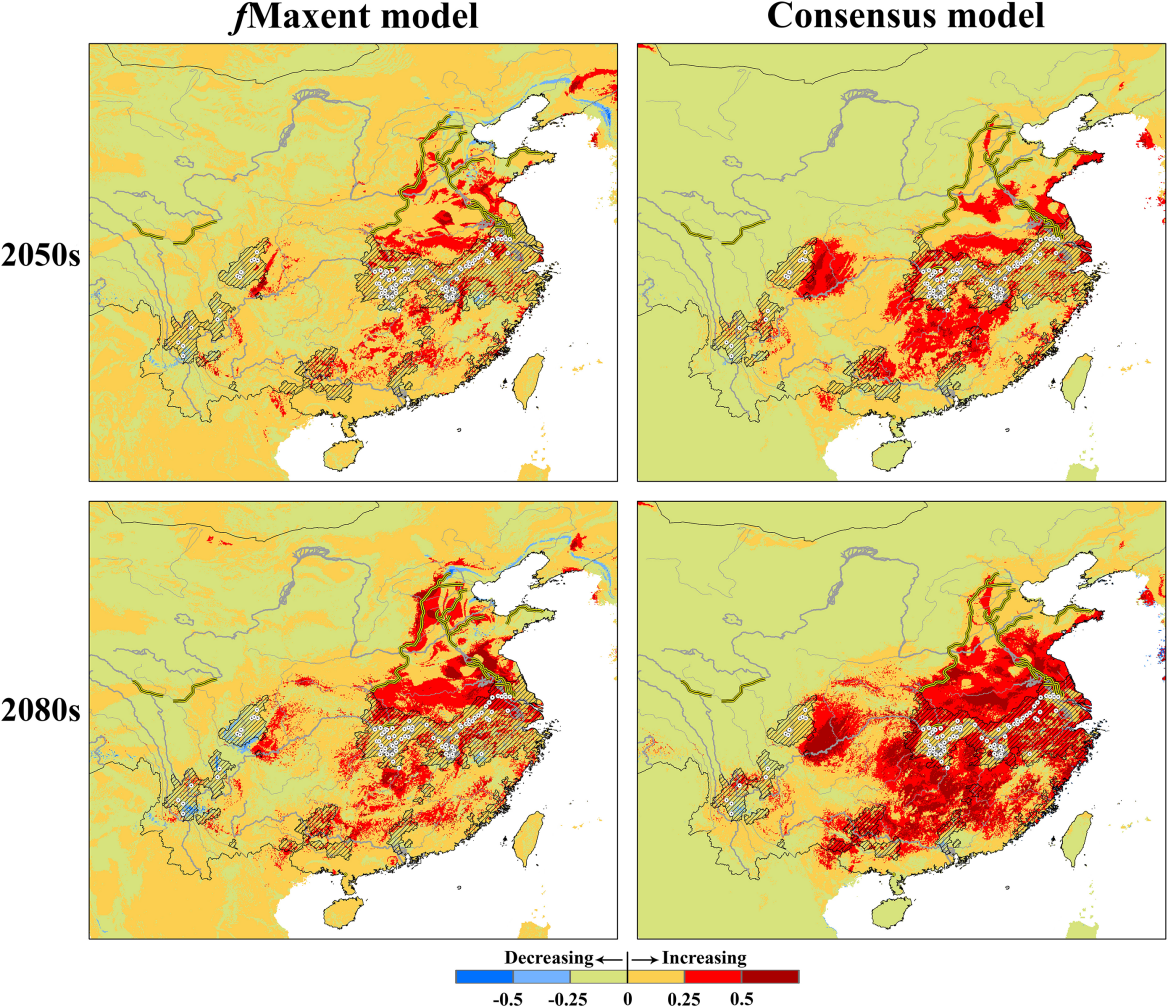
Predicted potential distributional changes for *Oncomelania hupensis* as a whole. The fine-tuned Maxent (*f*Maxent) and consensus models were calibrated on the accessible area and transferred onto future climate conditions in 2050–2060 (top) and 2080–2090 (bottom). White points indicate the surveillance sites, hatched areas indicate the administrative endemic areas.

**Fig 5:**
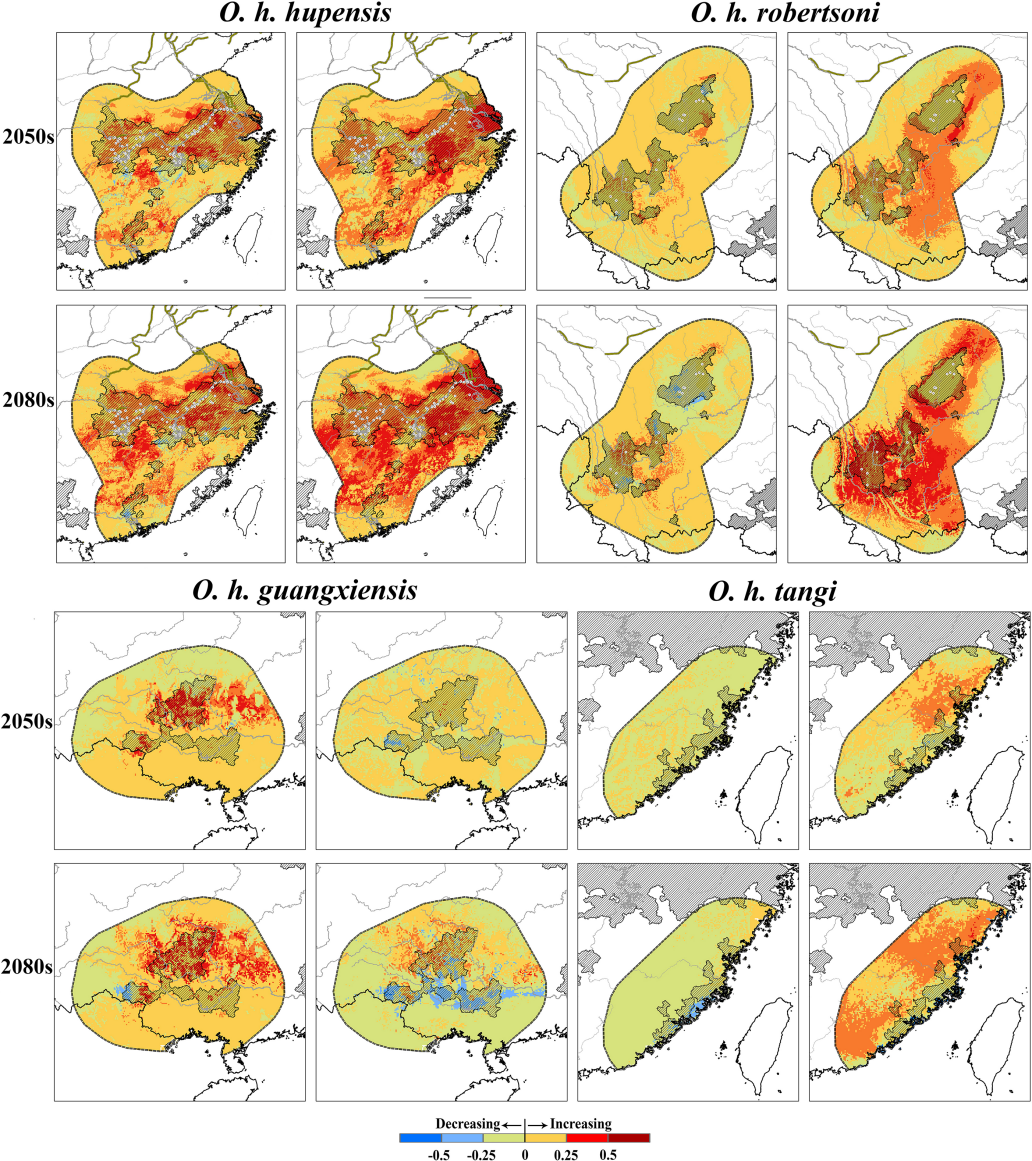
Potential distributional changes for the subspecies *Oncomelania hupensis hupensis* and *Oncomelania hupensis robertsoni*. The fine-tuned Maxent (left side) and consensus models (right side) were calibrated on the accessible areas and transferred onto future climate in 2050–2060 (up) and 2080–2090 (bottom). White points indicate the surveillance sites, hatched areas indicate the administrative endemic areas.

Variation among individual model predictions spatially was observed in both present and future (2050 and 2080; S5 Fig). Some areas identified as suitable nonetheless corresponded to environments beyond the climate envelope of the calibration area at present, thus involving non-analog climate conditions (S6 Fig). For example, *f*Maxent identified disjunct suitable areas around Beijing in northern China (Fig 4), but these areas involved model transfer into novel climate conditions (S6 Fig), making their interpretation uncertain and unwise. Within the distribution of each subspecies (Fig 1), projection of present ENMs onto future climate datasets generally involved little extrapolation (MOP metrics; S6 Fig). Transferring present-day models onto future climate scenarios, *f*Maxent models were more conservative than ensemble models (Figs 4 and 5): the western part of the predicted distribution based on consensus models was cleaner than predictions based on *f*Maxent, and the consensus method did not make the isolated predictions in the *f*Maxent model (Fig 4). Both *f*Maxent and consensus approaches identified a pattern of range expansion and suitability increase in *O*. *h*. *hupensis* (Figs 4 and 5). In *O*. *h*. *robertsoni*, both models identified an eastward shift, whereas in *O*. *h*. *guangxiensis*, a northward shift was indicated (Figs 4 and 5). In *O*. *h*. *tangi*, the two models showed contrasting predictions (Figs 4 and 5).

### SNWD and surveillance sites

**Fig 6:**
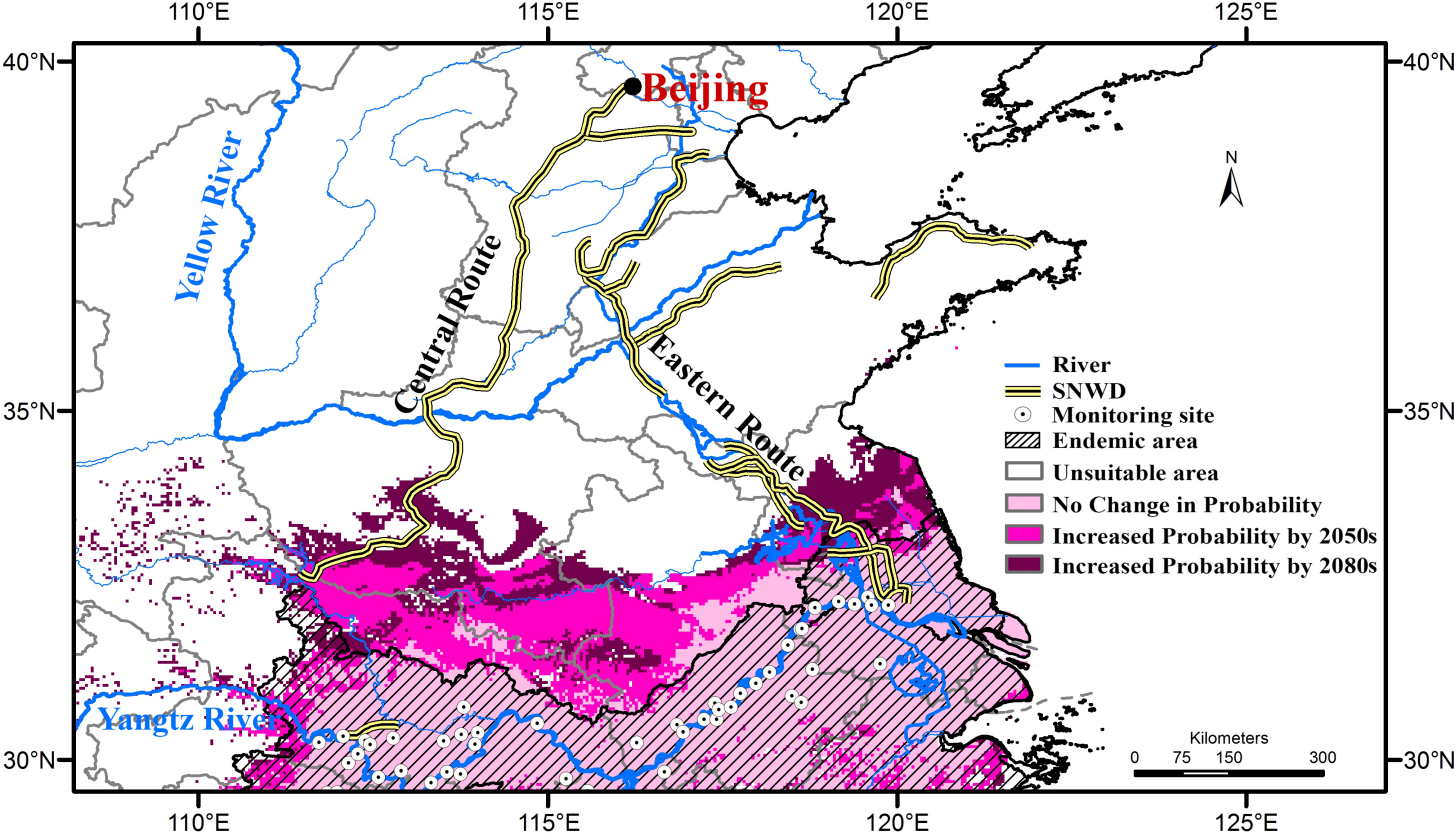
Future transmission potential of schistosomiasis projected on the Central Route and Eastern Route of the South-to-North-Water-Diversion project. Areas of agreement of *f*Maxent binary predictions of *Oncomelania hupensis* (as a whole) and *O*. *h*. *hupensis* are overlaid on the routes.

Binary predictions were based on *f*Maxent outputs, as thresholding future predictions from ensembles is difficult. Overlapping the Central Route and Eastern Route of SNWD with the binary future predictions for *O*. *hupensis* and *O*. *h*. *hupensis*, the southern Central Route coincides with suitable areas for *O*. *h*. *hupensis* in 2050–2060, and its suitable areas will expand northward along the southern Eastern Route by 2080–2090 (Fig 6). All of these areas are beyond the reach of present surveillance sites for schistosomiasis monitoring. Because a northward expansion of *O*. *hupensis* may occur considering future climate warming, these potential expansion areas need to be better covered by future surveillance efforts. Future surveillance efforts should also consider potential re-emergence of *O*. *h*. *guangxiensis* and *O*. *h*. *tangi*, as some areas of increasing suitability were noted for these two subspecies as well (Fig 5), although present intervention efforts have brought the snails to near extinction.

## Discussion

Limitations on materials and methodologies employed in this study need to be addressed here. While this paper focused on climate drivers, these factors occur in a complex milieu of other non-climatic drivers of snail distribution and parasite endemicity [21,22], although the non-climatic factors usually functioned at a small scale. Although we adopted ensemble forecasting approach to minimize the uncertainty of individual model predictions, the uncertainty exists in consensus models [29]. Ecological niche conservatism is of increasing importance given the complex impacts of ongoing climate change on biodiversity [4,6]. Many studies have evaluated niche conservatism across diverse evolutionary time spans [4,6]. Future projections for species involved in disease transmission and likely to respond to climate change are usually fraught with uncertainties and complexities; however, these assessments are crucial in identifying appropriate mitigation strategies [26]. Here, we tested climatic niche conservatism among the four subspecies of *O*. *hupensis* across mainland China, and integrated state-of-the-art modelling techniques (*f*Maxent and ensemble models) to forecast climate change effects. Our results have important implications regarding genetic divergence of *O*. *hupensis* and likely climate change effects on schistosomiasis transmission in mainland China.

The ecological niches of the four subspecies of *O*. *hupensis* were not identical, but we were unable to reject the null hypothesis that climatic niches are similar (except *O*. *h*. *robertsoni* versus *O*. *h*. *tangi*). Although failure to reject the null hypothesis does not assure that the climatic niche has been conserved, no evidence indicates that they have not been conserved, and broad climate spaces overlapped among the four subspecies (Fig 2 and S2 Fig). The relationship between niche overlap and phylogenetic relationships of the four subspecies further supports the idea that climate niches have been conserved (Fig 2 and S3 Fig). The signal of climate niche conservatism suggests that factors other than climate likely account for the genetic divergence of *O*. *hupensis* populations. Li et al. suggested that genetic differentiation of *O*. *hupensis* in mainland China is ultimately structured by landscape ecology [18], with populations falling into four different ecological settings (Fig 1): swamps and lakes in the Yangtze River Basin (*O*. *h*. *hupensis*); the mountainous region of Sichuan and Yunnan Provinces (*O*. *h*. *robertsoni*); the hilly, littoral part of Fujian province (*O*. *h*. *tangi*); and the karst landscape of Guangxi Autonomous Region (*O*. *h*. *guangxiensis*). This landscape-level segmentation of the four subspecies is generally consistent with the foundational work of Liu et al. [14]: indeed, clear geographic barriers separate the four subspecies (Fig 1; [14,16]). Climate niche divergence between *O*. *h*. *robertsoni* and *O*. *h*. *tangi* might relate to the long geographic distance separating them.

Previous studies have found that long-term climate warming tends to favor geographic expansion of *S*. *japonicum* in mainland China, but most such risk assessments have relied solely on mechanistic approaches (e.g. [23,25,48]). Although mechanistic models may be more desirable in that they estimate dimensions of the fundamental niche and in that they avoid problems with extrapolation [49], correlative ENMs have practical advantages in terms simplicity and flexibility, particularly as regards parameterization [50]. Comparing with mechanistic models, which predict a broad northward and westward expansion of *S*. *japonica* [23,25,48], correlative ENMs suggest a similar pattern, but with more detailed spatial predictions. Increased suitability and range expansion were observed consistently in *O*. *h*. *hupensis*, eastward and northward shifts in *O*. *h*. *robertsoni* and *O*. *h*. *guangxiensis*, and relatively stability status in *O*. *h*. *gangi* were observed in all our future model predictions (Figs 4 and 5).

Most current surveillance sites are distributed along the Yangtze River, designated to monitor transmission by *O*. *h*. *hupensis* and *O*. *h*. *robertsoni*. However, in a climate change context, both of these subspecies are expected to expand or shift distributionally (Fig 5). Surveillance sites distribution will have to broaden in coverage to be able to detect these shifts. In addition, the potential of *O*. *h*. *guangxiensis* and *O*. *h*. *tangi* to re-remerge should also be considered, as sites presenting increased suitability were identified (Fig 5). The southern parts of the Central Route of South-to-North Water Diversion (SNWD) project will become suitable for *O*. *h*. *hupensis* in 2050–2060, and suitable areas will expand northward along the southern parts of the Eastern Route of SNWD by 2080–2090: these areas are not covered by present surveillance efforts (Fig 6). Our results call for more rigorous monitoring and surveillance of schistosomiasis in the northern of potential expansion areas, although schistosomiasis currently has not been detected along either the southern Central Route or the Eastern Route; nonetheless, range expansion may open potential for emergence [48,51].

## Supporting information

S1 Table Bioclimatic variables used in niche models and future projections for *Oncomelania hupensis* and its subspecies across mainland China. (DOCX)

S2 Table Details and settings for each model algorithm used to fit individual niche models. (DOCX)

S3 Table Thirteen climate model projections and a short description of each used in the mathematical ensemble of future climate conditions for East Asia. (DOCX)

S4 Table Variance projection of individual climate models on the first principal component obtained by conducting a principal components analysis (PCA) on the 13 climate models. The bold values underline the six models for which PC1s were highest; these six modelling techniques were selected for the “PCA(median)” consensus method. Climate model abbreviations refer to S1 Table.
(DOCX)

S1 Fig Variation among 13 climate models in 8 climate dimensions for 2050 and 2080. Warm-colored areas indicate high variation among individual climate models. Bioclimatic variable abbreviations refer to S1 Table.
(DOCX)

S2 Fig Violin plots of bioclimatic variables occupied by the four subspecies of *Oncomelania hupensis*. Bioclimatic variable abbreviations refer to S1 Table.
(DOCX)

S3 Fig Climate niche identity and similarity test in each pair of the four subspecies. Red diamonds indicate actual niche overlap distributed in the frequency of simulated overlaps (gray volume), * indicates the significance of niche identity and similarity tests.
(DOCX)

S4 Fig AICc metrics for Maxent models among multiple settings. Maxent models were run with regularization multipliers ranging from 0.5 to 6.0 (increments of 0.5) using six different feature combinations (L, LQ, H, LQH, LQHP, LQHPT; where L = linear, Q = quadratic, H = hinge, P = product and T = threshold). Black arrows denote the AICc-selected models (i.e. minimum AICc values where delta AICc = 0). AICc-selected models settings are given in each box, together with performance metrics. Models were generated for the four subspecies separately, and for *Oncomelania hupensis* as a whole.
(DOCX)

S5 Fig Spatial variation of individual niche model performance across present and future models (2050–2060 and 2080–2090) for the four subspecies and for *Oncomelania hupensis* as a whole. Warm colors indicate high variation of individual model predictions.
(DOCX)

S6 Fig Mobility-oriented parity (MOP) assessment of environments of areas with different degree of similarity. Areas with different degree of similarity (grays) and strict extrapolation (black) were assessed between present and future climate datasets for the four subspecies and *Oncomelania hupensis* as a whole.
(DOCX)

S1 File The trimed occurrenced points used for climate space comparisons and model predictions. (CSV)
