# Supplementary material for: Schistosoma japonicum transmission risk maps at present and under climate change in mainland China
Source: PLoS Negl Trop Dis. 2017 Oct 17;11(10):e0006021. doi: 10.1371/journal.pntd.0006021 (PMC5659800; doi:10.1371/journal.pntd.0006021)
Supplement: S1 Table — (DOCX) [file pntd.0006021.s001.docx]

**S1 Table.** Bioclimatic variables used in niche models and future projections for *Oncomelania hupensis* and its subspecies across mainland China.

| Variable | Description |
| --- | --- |
| bio1 | Annual mean temperature |
| bio2 | Mean diurnal range (i.e., mean of monthly (maximum temperature – minimum temperature)) |
| bio3 | Isothermality (i.e., mean diurnal range / (maximum temperature of warmest month – minimum temperature of coldest month) |
| bio4 | Temperature standard deviation (i.e., seasonality) |
| bio10 | Mean temperature of warmest quarter |
| bio12 | Annual precipitation |
| bio14 | Precipitation of driest month |
| bio15 | Precipitation coefficient of variation (i.e., seasonality) |
