# Supplementary material for: Schistosoma japonicum transmission risk maps at present and under climate change in mainland China
Source: PLoS Negl Trop Dis. 2017 Oct 17;11(10):e0006021. doi: 10.1371/journal.pntd.0006021 (PMC5659800; doi:10.1371/journal.pntd.0006021)
Supplement: S2 Table — (DOCX) [file pntd.0006021.s002.docx]

**S2 Table.** Details and settings for each model algorithm used to fit individual niche models.

| Model | Platform | Parameters |
| --- | --- | --- |
| GAM | BIOMOD2 | 10000 of random pseudo-absence records, presence and absence equal weight, algo = 'GAM_mgcv', type = 's_smoother', *k* = -1, family = binomial (link = 'logit'), method = 'GCV.Cp', optimizer = c ('outer','newton'), others default. |
| GBM | BIOMOD2 | Random pseudo-absence records, equal number of presence records, presence and absence equal weight, distribution = 'bernoulli', n.trees = 2000, interaction.depth = 7, n.minobsinnode = 5, shrinkage = 0.001, bag.fraction = 0.5, train.fraction = 1, cv.folds = 3, others default. |
| GLM | BIOMOD2 | 10000 of random pseudo-absence records, presence and absence equal weight, type = 'polynomial', interaction.level = 0, test = 'AIC', family = binomial (link = 'logit'), mustart = 0.5, others default. |
| RF | BIOMOD2 | Random pseudo-absence records, equal number of presence records, presence and absence equal weight, do.classif = T, Ntree = 500, nodesize = 5, others default. |
| GARP | desktop GARP 1.1.3 | Maximum iterations 1000, best subset approach (100 runs, 20 models of lowest omission selected, within which 10 models of medium commission error reserved), others default. |
| *f*Maxent | Maxent 3.3.3k | Logistic output, maximum iteration = 500, Features and regularization multiplier values for each species refer to S8 Fig, others default. |
