# Supplementary material for: Schistosoma japonicum transmission risk maps at present and under climate change in mainland China
Source: PLoS Negl Trop Dis. 2017 Oct 17;11(10):e0006021. doi: 10.1371/journal.pntd.0006021 (PMC5659800; doi:10.1371/journal.pntd.0006021)
Supplement: S3 Table — (DOCX) [file pntd.0006021.s003.docx]

**S3 Table.** Thirteen climate model projections and a short description of each used in the mathematical ensemble of future climate conditions for East Asia.

| Model Name | | Abb. | | Full name, version, and location |
| --- | --- | --- | --- | --- |
| ACCESS 1.0 | AC | | Australian Community Climate and Earth-System Simulator Model, Version 1.0, Australia | |
| BCC-CSM1.1 | BC | | Beijing Climate Center Climate System Model, Version1.1, China | |
| CCSM4 | CC | | Community Climate System Model, Version 4, USA | |
| GFDL-CM3 | GF | | Geophysical Fluid Dynamics Laboratory Climate Model, Version 3, USA | |
| GISS-E2-R | GS | | Goddard Institute for Space Studies General Circulation Model, version ModelE2, USA | |
| HadGEM2-ES | HE | | Hadley Centre Global Environmental Model, Version 2, United Kingdom | |
| INMCM4 | IN | | Institute of Numerical Mathematics Climate Model, Version 4, Russia | |
| IPSL-CM5A-LR | IP | | Institut Pierre Simon Laplace Climate Model, version CM5A, low resolution, France | |
| MIROC5 | MC | | Model for Interdisciplinary Research On Climate, Version 5, Japan | |
| MRI-CGCM3 | MG | | Meteorological Research Institute Coupled Global Climate Model, Version 3, Japan | |
| MPI-ESM-LR | MP | | Max Planck Institute Earth System Model, Version low resolution, Germany | |
| MIROC-ESM | MR | | Model for Interdisciplinary Research On Climate Earth System Model, version 2010, Japan | |
| NorESM1-M | NO | | Norwegian Earth System Model, version intermediate resolution, Norway | |
