# Supplementary material for: Schistosoma japonicum transmission risk maps at present and under climate change in mainland China
Source: PLoS Negl Trop Dis. 2017 Oct 17;11(10):e0006021. doi: 10.1371/journal.pntd.0006021 (PMC5659800; doi:10.1371/journal.pntd.0006021)
Supplement: S4 Table — The bold values underline the six models for which PC1s were highest; these six modelling techniques were selected for the “PCA(median)” consensus method. Climate model abbreviations refer to S1 Table. (DOCX) [file pntd.0006021.s004.docx]

**S4 Table.** Variance projection of individual climate models on the first principal component obtained by conducting a principal components analysis (PCA) on the 13 climate models. The bold values underline the six models for which PC1s were highest; these six modelling techniques were selected for the “PCA(median)” consensus method. Climate model abbreviations refer to S1 Table.

| Models | AC | BC | CC | GF | GS | HE | IN | IP | MC | MG | MP | MR | NO |
| --- | --- | --- | --- | --- | --- | --- | --- | --- | --- | --- | --- | --- | --- |
| 2050s |  |  |  |  |  |  |  |  |  |  |  |  |  |
| bio1 | 0.275 | **0.283** | **0.277** | 0.271 | 0.275 | **0.284** | **0.280** | 0.275 | 0.273 | **0.284** | **0.281** | 0.271 | 0.277 |
| bio2 | 0.258 | 0.273 | **0.277** | **0.310** | **0.284** | 0.271 | **0.289** | 0.275 | 0.277 | **0.278** | **0.280** | 0.252 | 0.276 |
| bio3 | **0.284** | 0.270 | 0.274 | 0.275 | **0.284** | 0.269 | **0.278** | **0.289** | 0.275 | 0.274 | **0.289** | 0.263 | **0.279** |
| bio4 | **0.285** | 0.276 | 0.274 | **0.286** | 0.270 | 0.271 | 0.270 | **0.279** | **0.281** | 0.271 | 0.277 | **0.283** | **0.281** |
| bio10 | **0.280** | **0.281** | 0.276 | 0.266 | 0.271 | **0.285** | **0.282** | 0.279 | 0.268 | **0.286** | **0.281** | 0.273 | 0.277 |
| bio12 | **0.279** | **0.284** | **0.285** | **0.279** | 0.277 | 0.276 | 0.268 | 0.270 | **0.280** | 0.272 | 0.276 | 0.275 | **0.286** |
| bio14 | **0.298** | 0.261 | 0.262 | 0.253 | **0.302** | **0.288** | **0.295** | 0.223 | **0.326** | 0.265 | **0.312** | 0.249 | 0.253 |
| bio15 | **0.305** | 0.263 | 0.256 | 0.268 | 0.269 | **0.283** | **0.290** | **0.278** | **0.277** | 0.270 | 0.263 | **0.307** | 0.271 |
| 2080s |  |  |  |  |  |  |  |  |  |  |  |  |  |
| bio1 | 0.275 | **0.284** | **0.281** | 0.269 | 0.276 | **0.285** | **0.282** | 0.275 | 0.273 | **0.285** | **0.278** | 0.269 | 0.274 |
| bio2 | 0.241 | 0.273 | 0.264 | **0.316** | **0.285** | 0.276 | **0.286** | **0.284** | 0.274 | **0.281** | **0.285** | 0.265 | 0.272 |
| bio3 | **0.299** | 0.270 | **0.282** | 0.256 | **0.300** | **0.281** | **0.286** | 0.279 | **0.280** | 0.263 | 0.270 | 0.279 | 0.256 |
| bio4 | 0.277 | **0.279** | 0.277 | 0.275 | **0.283** | **0.278** | 0.275 | 0.275 | **0.285** | 0.268 | **0.282** | **0.286** | 0.264 |
| bio10 | **0.281** | **0.283** | **0.279** | 0.266 | 0.270 | **0.290** | 0.279 | **0.281** | 0.269 | **0.283** | 0.277 | 0.272 | 0.274 |
| bio12 | **0.294** | 0.274 | 0.273 | 0.268 | **0.285** | **0.283** | 0.273 | 0.269 | **0.280** | **0.282** | 0.274 | 0.263 | **0.286** |
| bio14 | 0.271 | 0.221 | 0.254 | 0.251 | **0.360** | **0.296** | 0.207 | 0.177 | 0.258 | **0.297** | **0.320** | **0.335** | **0.300** |
| bio15 | **0.284** | **0.288** | 0.240 | 0.261 | **0.308** | 0.274 | 0.261 | **0.306** | 0.252 | 0.268 | **0.279** | **0.308** | 0.266 |
