# Supplementary material for: Schistosoma japonicum transmission risk maps at present and under climate change in mainland China
Source: PLoS Negl Trop Dis. 2017 Oct 17;11(10):e0006021. doi: 10.1371/journal.pntd.0006021 (PMC5659800; doi:10.1371/journal.pntd.0006021)
Supplement: S1 Fig — Warm-colored areas indicate high variation among individual climate models. Bioclimatic variable abbreviations refer to S1 Table. (DOCX) [file pntd.0006021.s005.docx]

**S1 Fig.** Variation among 13 climate models in 8 climate dimensions for 2050 and 2080. Warm-colored areas indicate high variation among individual climate models. Bioclimatic variable abbreviations refer to S1 Table.

**
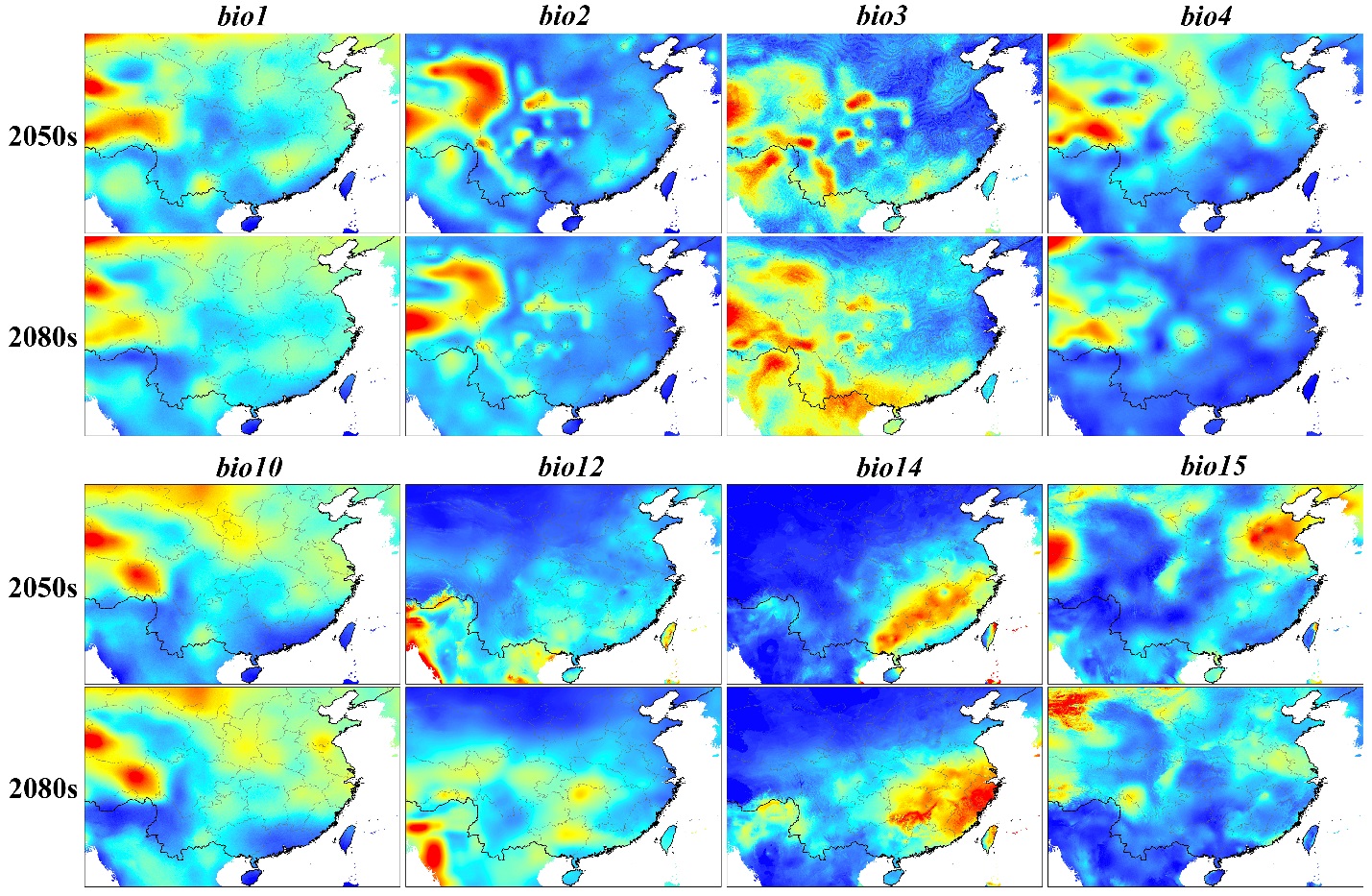
**
