# Supplementary material for: Schistosoma japonicum transmission risk maps at present and under climate change in mainland China
Source: PLoS Negl Trop Dis. 2017 Oct 17;11(10):e0006021. doi: 10.1371/journal.pntd.0006021 (PMC5659800; doi:10.1371/journal.pntd.0006021)

**S2 Fig.** Violin plots of bioclimatic variables occupied by the four subspecies of *Oncomelania hupensis*. Bioclimatic variable abbreviations refer to S1 Table.


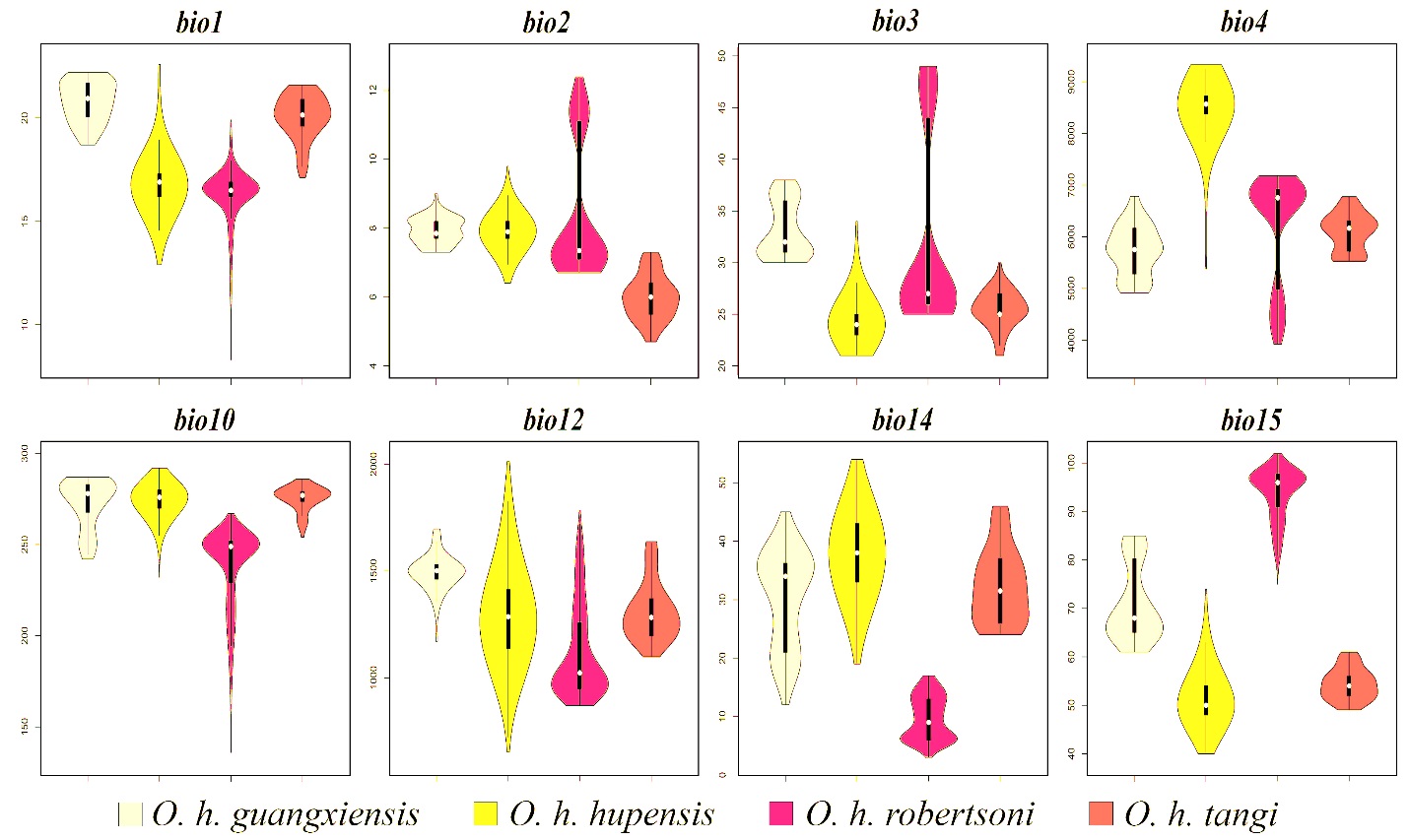

Supplement: S2 Fig — Bioclimatic variable abbreviations refer to S1 Table. (DOCX) [file pntd.0006021.s006.docx]
