# Supplementary material for: Schistosoma japonicum transmission risk maps at present and under climate change in mainland China
Source: PLoS Negl Trop Dis. 2017 Oct 17;11(10):e0006021. doi: 10.1371/journal.pntd.0006021 (PMC5659800; doi:10.1371/journal.pntd.0006021)

**S3 Fig.** Climate niche identity and similarity test in each pair of the four subspecies. Niche identity test determines whether niches of two subspecies in two geographical ranges are equivalent (i.e. whether the niche overlap is constant when randomly reallocating the occurrences of both subspecies among the two ranges), whereas niche similarity test addresses whether the environmental niche occupied in one range is more similar to the one occupied in the other range than would be expected by chance. Red diamonds indicate actual niche overlap distributed in the frequency of simulated overlaps (gray volume), * indicates the significance of niche identity and similarity tests.


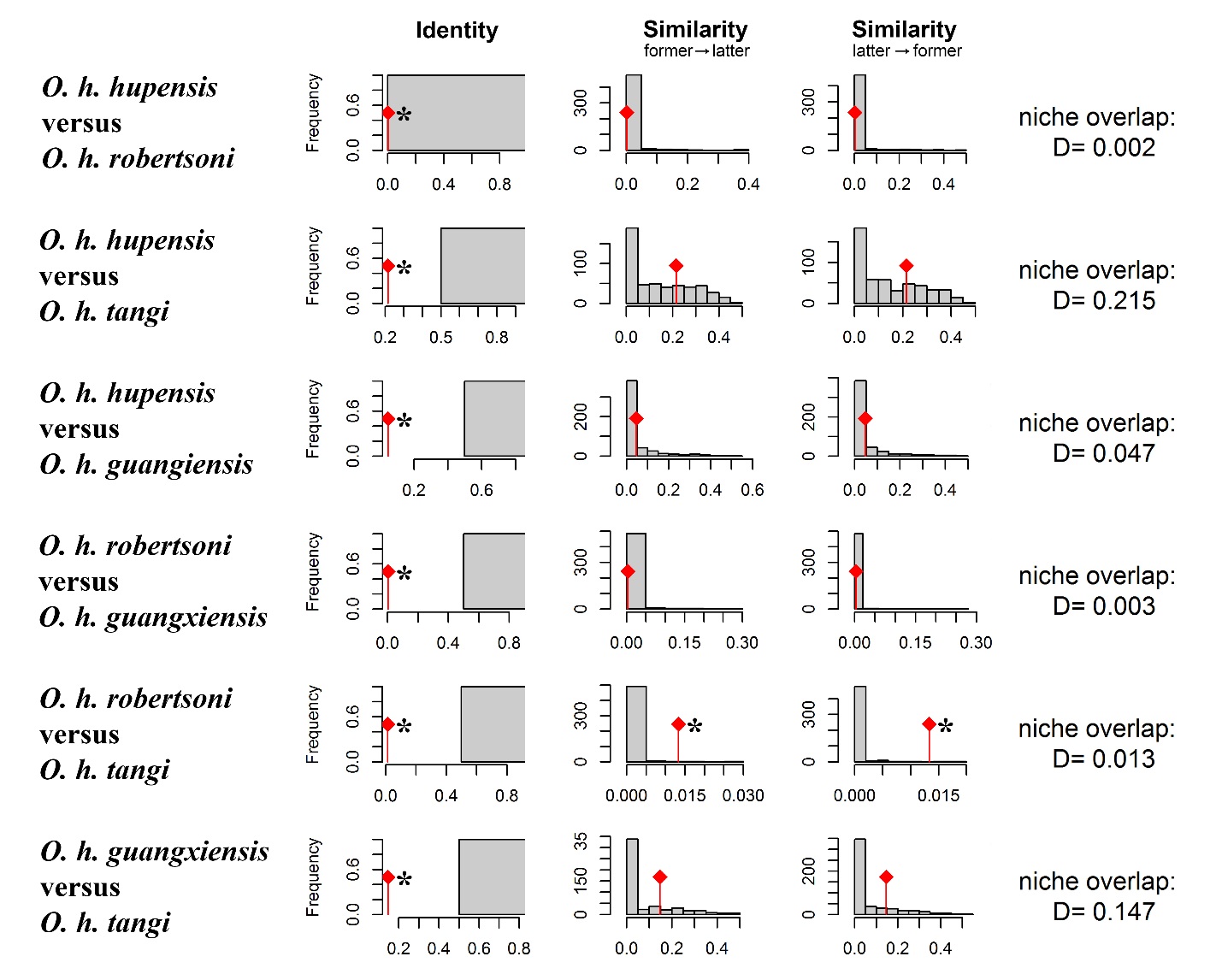

Supplement: S3 Fig — Red diamonds indicate actual niche overlap distributed in the frequency of simulated overlaps (gray volume), * indicates the significance of niche identity and similarity tests. (DOCX) [file pntd.0006021.s007.docx]
