# Supplementary material for: Schistosoma japonicum transmission risk maps at present and under climate change in mainland China
Source: PLoS Negl Trop Dis. 2017 Oct 17;11(10):e0006021. doi: 10.1371/journal.pntd.0006021 (PMC5659800; doi:10.1371/journal.pntd.0006021)
Supplement: S4 Fig — Maxent models were run with regularization multipliers ranging from 0.5 to 6.0 (increments of 0.5) using six different feature combinations (L, LQ, H, LQH, LQHP, LQHPT; where L = linear, Q = quadratic, H = hinge, P = product and T = threshold). Black arrows denote the AICc-selected models (i.e. minimum AICc values where delta AICc = 0). AICc-selected models settings are given in each box, together with performance metrics. Models were generated for the four subspecies separately, and for Oncomelania hupensis as a whole. (DOCX) [file pntd.0006021.s008.docx]

**S4 Fig.** AICc metrics for Maxent models among multiple settings. Maxent models were run with regularization multipliers (RM) ranging from 0.5 to 6.0 (increments of 0.5) using six different feature combinations (FC: L, LQ, H, LQH, LQHP, LQHPT; where L = linear, Q = quadratic, H = hinge, P = product and T = threshold). Black arrows denote the AICc-selected models (i.e. minimum AICc values where delta AICc = 0). ENMeval automatically executes Maxent across a range of FCs and RMs settings, providing several evaluation metrics to aid in identifying settings that balance model goodness-of-fit with model complexity. RM acts in concert across all FCs as a coefficient multiplied to the individual regularization values that correspond to each respective FC. AICc-selected models settings are given in each box, together with performance metrics. The model with the lowest AICc value (i.e. delta. AICc = 0) is considered the best model out of the current suite of models. Models were generated for the four subspecies separately, and for *Oncomelania hupensis* as a whole.

**
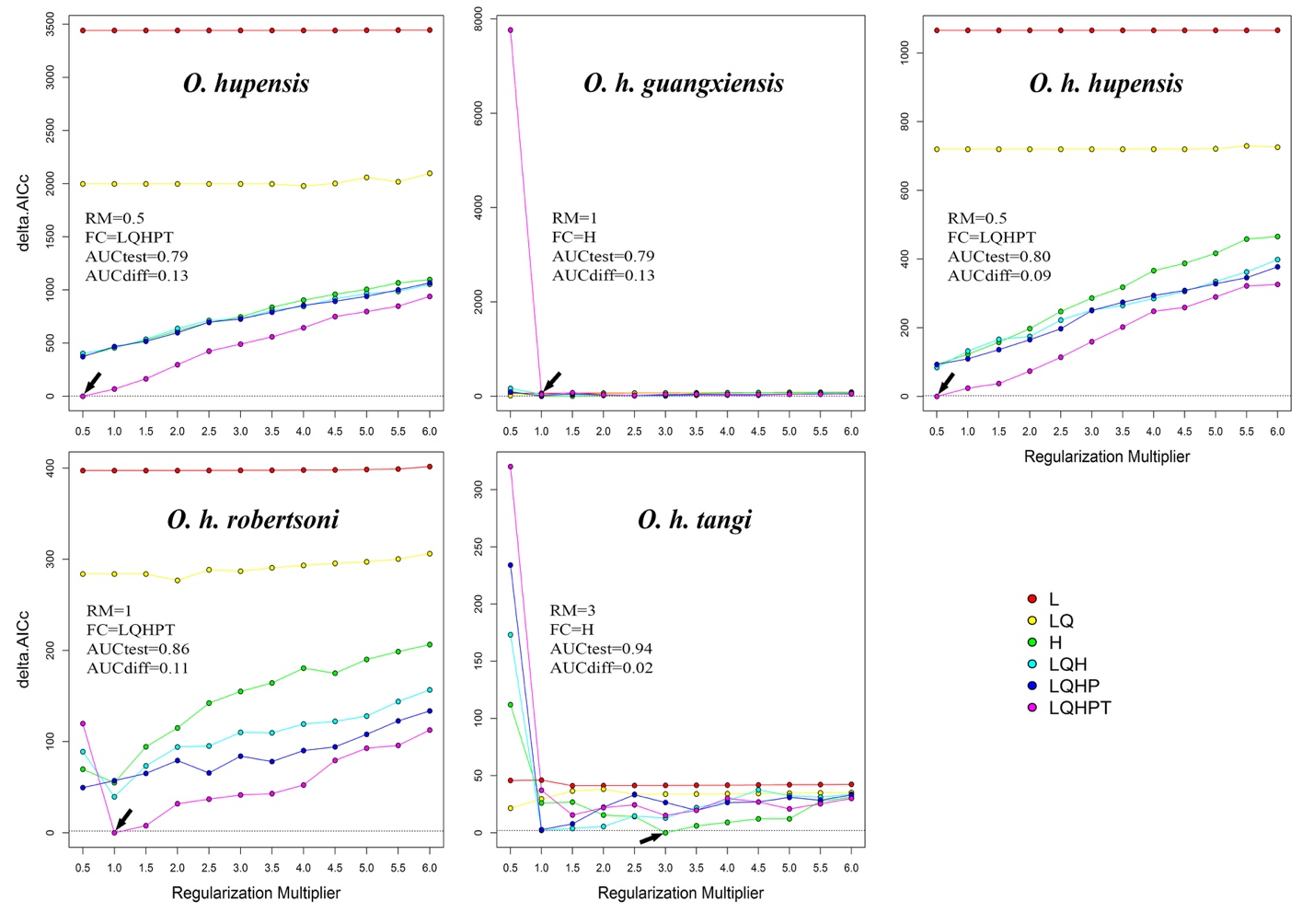
**
