# Supplementary material for: Schistosoma japonicum transmission risk maps at present and under climate change in mainland China
Source: PLoS Negl Trop Dis. 2017 Oct 17;11(10):e0006021. doi: 10.1371/journal.pntd.0006021 (PMC5659800; doi:10.1371/journal.pntd.0006021)

**S5 Fig.** Spatial variation of individual niche model performance across present and future models (2050-2060 and 2080-2090) for the four subspecies and for *Oncomelania hupensis* as a whole**.** Warm colors indicate high variation of individual model predictions.


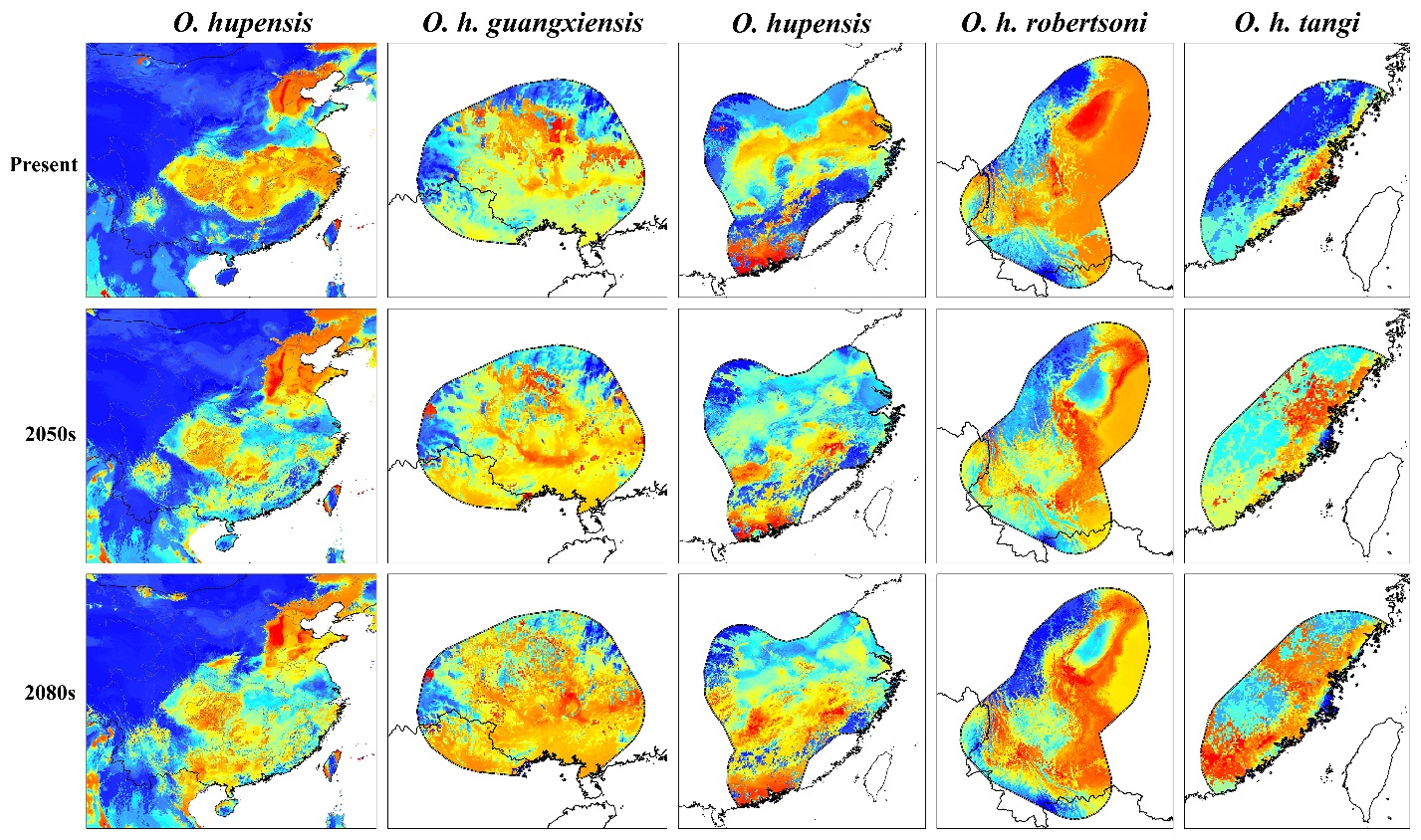

Supplement: S5 Fig — Warm colors indicate high variation of individual model predictions. (DOCX) [file pntd.0006021.s009.docx]
