# Supplementary material for: Schistosoma japonicum transmission risk maps at present and under climate change in mainland China
Source: PLoS Negl Trop Dis. 2017 Oct 17;11(10):e0006021. doi: 10.1371/journal.pntd.0006021 (PMC5659800; doi:10.1371/journal.pntd.0006021)

**S6 Fig.** Mobility-oriented parity (MOP) assessment of environments of areas with different degree of similarity. Areas with different degree of similarity (grays) and strict extrapolation (black) were assessed between present and future climate datasets for the four subspecies and *Oncomelania hupensis* as a whole. Projections of present ENMs onto future climate datasets generally involved little extrapolation.


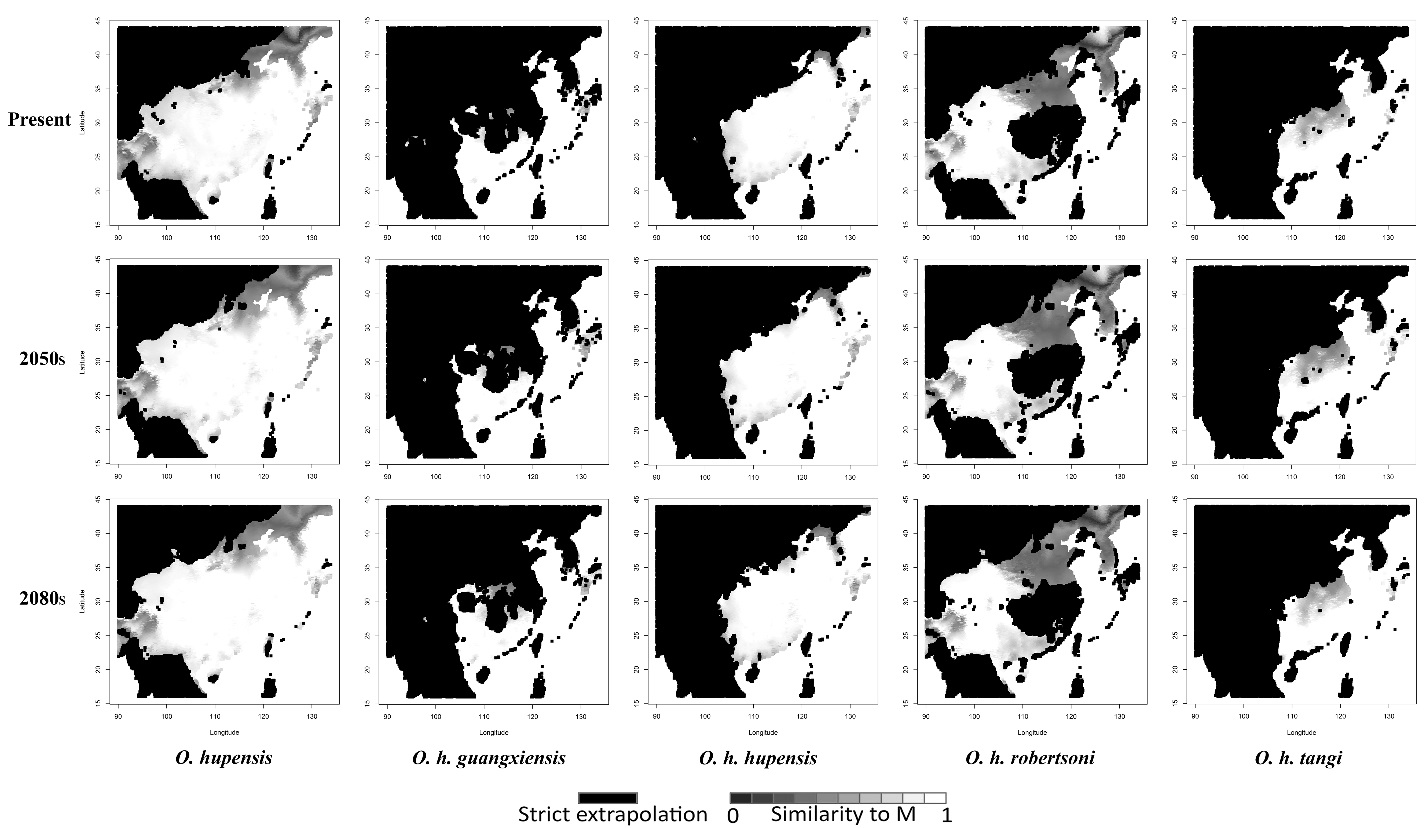

Supplement: S6 Fig — Areas with different degree of similarity (grays) and strict extrapolation (black) were assessed between present and future climate datasets for the four subspecies and Oncomelania hupensis as a whole. (DOCX) [file pntd.0006021.s010.docx]
